# Revisiting MOF-Derived Single-Atom Electrocatalysts: Limitations, Characterizations, and Design Strategies

**DOI:** 10.1021/acs.nanolett.5c05986

**Published:** 2026-01-23

**Authors:** Zheao Huang, Dominik Eder

**Affiliations:** Institute of Materials Chemistry, 164718 Technische Universität Wien, 1060 Vienna, Austria

**Keywords:** single-atom sites, metal−organic frameworks, ligand and defect engineering

## Abstract

Single-atom sites (SASs) and their electrocatalysts offer outstanding catalytic activity and metal efficiency. Metal–organic frameworks (MOFs), with their tunable and multifunctional architectures, serve as ideal precursors for SASs, enabling atomic-level dispersion. However, current research often overlooks critical ambiguities in SAS definitions, intrinsic limitations, and characterization reliability. Moreover, prevalent destructive treatments, such as pyrolysis or sulfidation, inevitably compromise framework integrity, raising concerns regarding the trade-off between structural designability and conductivity. Accordingly, this Mini-Review critically revisits MOF-derived SASs by scrutinizing synthesis limitations and emphasizing the quantitative assessment of atomic utilization efficiency. Representative examples of emerging framework-retaining strategies, including ligand and defect engineering, are discussed to illustrate opportunities for preserving MOF advantages. Finally, future directions are proposed, focusing on dynamic structural reconstruction and operando validation to simultaneously enhance activity, stability, and scalability for practical energy conversion applications.

## Graphical abstract

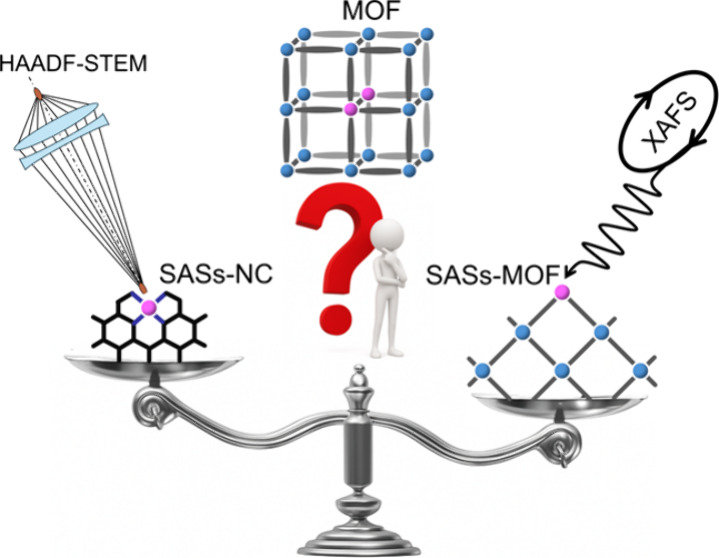

## Introduction

Single-atom sites (SASs) and their corresponding single-atom catalysts (SACs) are typically defined as metal atoms atomically dispersed in an isolated manner on a support surface.^1^ In principle, two essential criteria must be met: the metal–metal distance must be sufficiently large to ensure true isolation, and nearly 100% atomic utilization should be achieved (Figure 1a). Compared with cluster- or nanoparticulate-counterparts, SASs generally exhibit superior activity and/or selectivity in various reactions.^2^ In catalysis and related energy fields, SASs have attracted immense attention because they can minimize noble-metal usage while maximizing both specific (turnover frequency) and mass (metal utilization efficiency) activities.^3^ Consequently, the number of studies on SASs/SACs has increased exponentially over the past decade, particularly in electrocatalysis. Most reported systems employ porous metal–organic frameworks (MOFs) or other carbon-based materials as supports.^4,5^ Typically, noble metals or first-row transition metals (e.g., Ni, Fe, Co, and Cu) are introduced into the precursors and then converted into isolated SASs. Advanced characterization techniques, including synchrotron-based X-ray absorption fine structure (XAFS) spectroscopy and aberration-corrected high-angle annular dark-field scanning transmission electron microscopy (HAADF-STEM), are commonly used to verify atomic dispersion on both the average and local scales.

**Figure 1:**
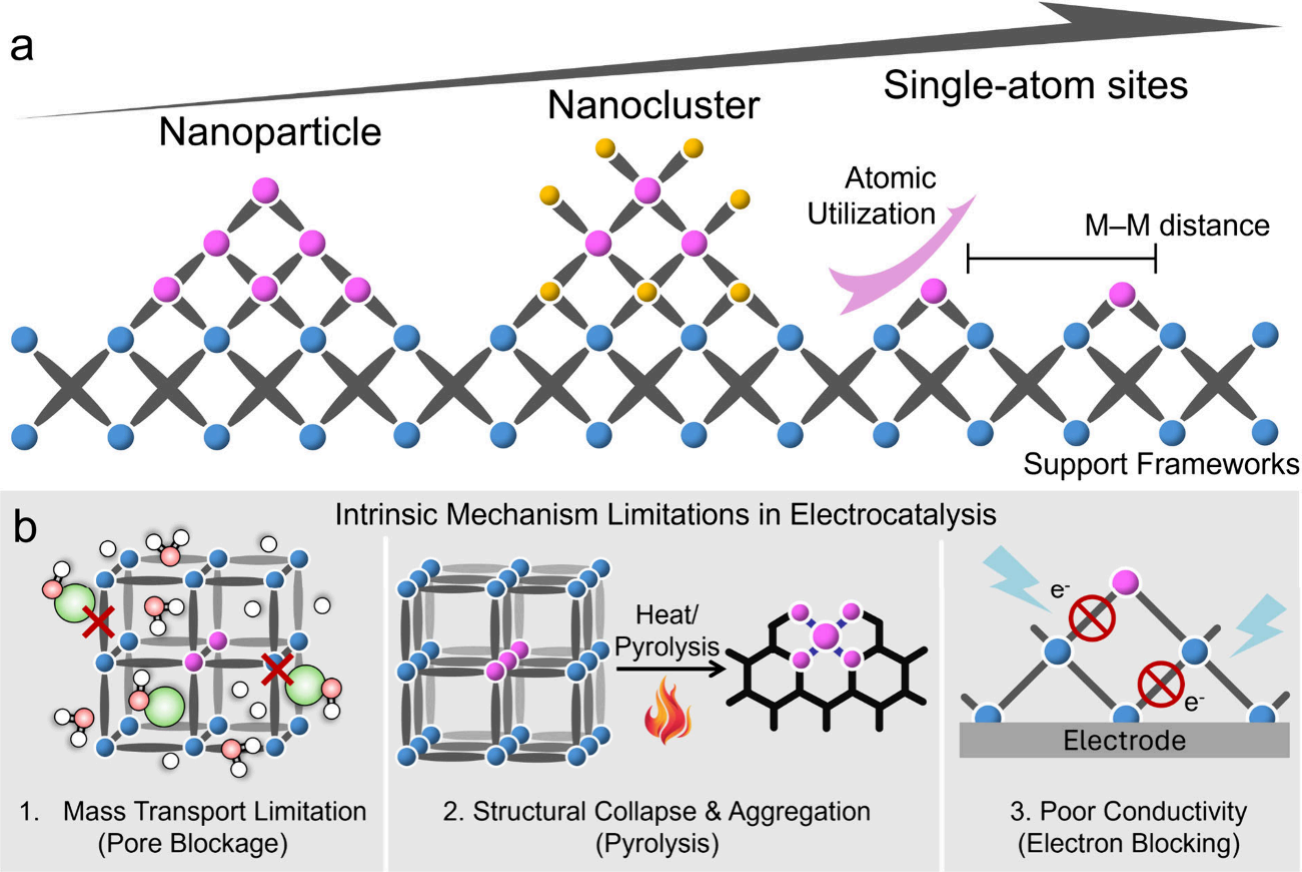
Conceptual definition and intrinsic limitations. (a) Schematic illustration of the structural evolution from nanoparticles (left) and nanoclusters (middle) to single-atom sites (right) within support frameworks, highlighting the differences in atomic utilization and metal–metal distance. (b) Schematic representation of the three critical intrinsic limitations of MOF-derived SASs in practical electrocatalysis. The gray connecting bonds are for representation only. Pink represents the target metal, blue denotes the framework nodes and/or secondary metals serving as supports, and yellow indicates potential coordination atoms for the target metal (such as N, Cl, S, and P).

The expanding interpretation of SASs, however, has led to increasingly ambiguous definitions and frequent misuse of characterization methods as the number of related reports continues to rise. While issues such as low metal loading and stability have been extensively reviewed,^6,7^ fundamental ambiguities regarding the strict definition of SASs remain unresolved. Two critical questions must be addressed: (1) What metal–metal distance truly qualifies a site as “single atomic,” and (2) how can one convincingly demonstrate that all isolated metal atoms are catalytically active and nearly 100% utilized? The first question can, in principle, be addressed using techniques such as XAFS and HAADF-STEM to verify sufficient metal–metal separation, typically greater than 5 Å. However, their results are often misapplied or misinterpreted in many current studies, leading some to claim them as the pinnacle methods for SAS characterization.^8,9^ The second question remains largely unexplored in most electrocatalytic research, as few studies investigated catalytic kinetics that directly support 100% active site utilization. This raises a critical issue: *How can these two defining features of SASs be more reliably validated using existing methods*?

Zeolitic imidazolate frameworks (ZIFs), a prominent subclass of MOFs, have emerged as preferred precursors for realizing atomic-level metal dispersion, owing to their facile synthesis, exceptional surface area, and abundant porosity.^10^ Typically, secondary metal species are impregnated into ZIF-8 (Zn), followed by high-temperature pyrolysis that volatilizes the native Zn, thereby generating atomically dispersed SASs anchored on nitrogen-doped carbon (NC). The periodic organic framework effectively prevents secondary metal aggregation and provides abundant nitrogen coordination environments for anchoring isolated atoms. However, this destructive treatment completely sacrifices the crystalline order and porosity of MOFs, raising a critical issue: *Is this MOF structural damage worth the gain in atomic dispersion*? Alternative strategies that retain the open-framework structures have also emerged; for example, Abdel-Mageed et al. summarized the postsynthetic metal exchange or incorporation of secondary metals directly into MOF matrices as SACs.^11^ However, these catalysts often feature bimetallic sites, defect-rich sites, and unsaturated/open metal sites (OMSs), which challenge the strict definition of true SASs. Moreover, their catalytic activity and stability are often inferior to those of NC-supported SACs due to the partial preservation of the organic framework, raising a further question: *Can the metal sites within these MOFs truly be regarded as single-atom sites*?

To address these challenges, this Mini-Review critically revisits MOF-derived single-atom electrocatalysts, specifically targeting the three fundamental bottlenecks illustrated in Figure 1b: mass transport hindrance in micropores, structural collapse during pyrolysis, and the trade-off between conductivity and active site accessibility. Distinct from prior reviews that primarily catalog synthetic routes or pyrolysis-derived structures,^12,13^ we shift the focus to a rigorous examination of methodological pitfalls. We clarify ambiguities in structural definitions and contrast destructive pyrolysis with emerging framework-retaining strategies. Furthermore, we highlight common misinterpretations in characterization (e.g., XAFS and HAADF-STEM), and advocate for the adoption of more robust diagnostic methodologies to definitively validate active moieties. Finally, we underscore the critical necessity of quantifying atomic utilization efficiency. By resolving these fundamental uncertainties, we aim to provide a clear roadmap for the rational design of next-generation MOF-based electrocatalysts that maximize both intrinsic activity and structural integrity.

## Structural Definition and Synthetic Strategies of MOF-Derived SASs

Before discussing the structural features of MOF-derived SASs/SACs, it is necessary to clarify one conceptual question: *Can a pristine MOF itself be considered a structure containing SASs*? This question arises naturally as the definition of SASs has become increasingly broad in recent years. Many MOFs, particularly those with mononuclear secondary building units (SBUs) containing only one metal center separated by long organic ligands, may appear to satisfy one criterion of SASs, namely a sufficiently large metal–metal distance. However, in practice, pristine MOFs are rarely regarded as SAS-containing structures. Ideally, pristine MOFs are rarely employed directly as electrocatalysts due to their inherent limitations: the insulating organic ligands impede charge transfer, while the excessively high metal content (15–40 wt %) and microporous bulk often render most metal nodes electrochemically inaccessible, failing to meet the criteria for atomic utilization efficiency. In contrast, 2D materials are often more effective in this regard. Although 2D conductive MOFs have recently been reported, their conduction mechanisms remain highly structure-sensitive, and their stability is still inferior to that of conventional materials.^14^ Therefore, based on the above reasons, most MOF-derived SASs are obtained not from the inherent metal centers of the MOF but by introducing a secondary metal through impregnation, ion exchange, ligand engineering or post- encapsulation, followed by anchoring the newly introduced atoms as true single-atom sites that participate directly in electrocatalysis.

Destructive thermal treatment stands as a dominant approach, leveraging mononuclear ZIF-8 as an ideal precursor due to its scalable synthesis and superior textural properties. Each SBU in ZIF-8 consists of a single Zn^2+^ ion coordinated to four 2-methylimidazolate (2-mIm) ligands through Zn–N bonds to form a Zn–N_4_ SBU.^15^ Upon partial substitution of Zn with a secondary metal, the M–N_4_ configurations are generated. Subsequent high-temperature pyrolysis (around 900 °C under inert gas) decomposes the organic ligands and evaporates the Zn species. This process yields atomically dispersed SASs anchored on nitrogen-doped carbon (NC) via M–N coordination, referred to here as NC-type SACs. Initially employing a spatial confinement strategy, Yu et al. successfully dispersed Rh precursor into ZIF-8 molecular cages through a host–guest strategy, followed by pyrolysis to obtain atomically dispersed SA-Rh/CN (Figure 2a), which exhibited promising electrocatalytic properties for formic acid oxidation.^16^ To further expose active sites beyond simple encapsulation, Li et al. subsequently prepared Mn-impregnated ZIF-8 followed by pyrolysis to remove Zn and acid etching, obtaining a porous Mn–N–C composite where isolated Mn sites served as highly efficient oxygen reduction reaction (ORR).^17^ Comparable strategy have been applied to synthesize Fe–, Co– and Pt–N–C catalysts, all demonstrating outstanding electrocatalytic activity.^18^ Other heteroatom-doped carbon materials, such as sulfur- or phosphorus-doped carbon, have also been reported as effective support for stabilizing single metal atoms.^19,20^ Introducing a secondary metal while converting the organic ligands into conductive materials effectively overcomes the intrinsic limitations of pristine MOFs when used directly as SACs. Moreover, the low secondary metal loading ensures sufficient metal–metal separation, suppressing aggregation during pyrolysis and enabling the formation of SASs. Compared with binuclear or multinuclear MOFs, such as the dinuclear paddle-wheel SBU in HKUST-1 or polynuclear SBU in UIO-66, mononuclear ZIFs are ideal precursors for constructing NC-type SACs via pyrolysis.

**Figure 2:**
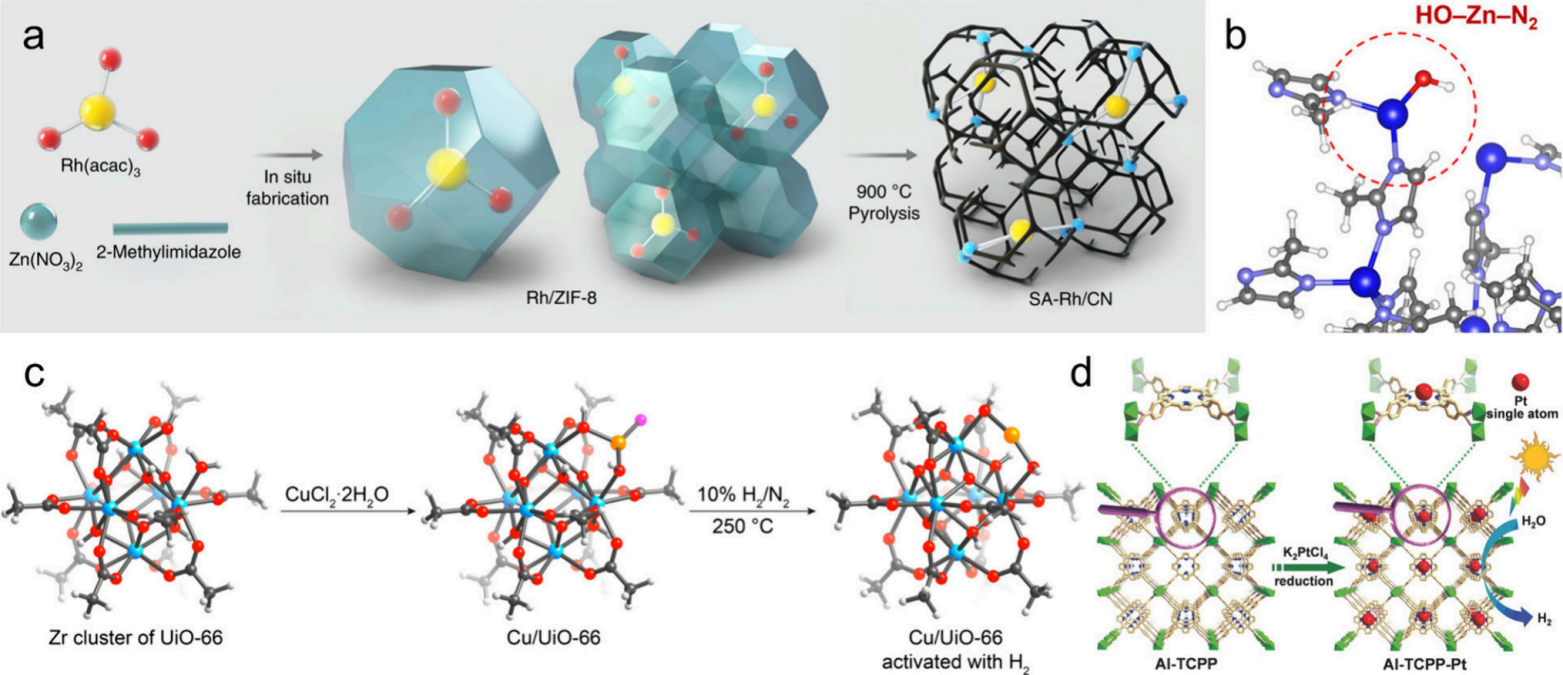
Representative synthetic strategies for MOF-derived SASs. (a) Preparation of SA-Rh/CN via spatial confinement followed by pyrolysis. Reproduced with permission from ref 16. Copyright 2020 Springer Nature. (b) Optimized HO–Zn–N_2_ active sites engineered within the ZIF-8 framework. Reproduced with permission from ref 22. Copyright 2024 Wiley-VCH GmbH. (c) DFT-calculated structures of Cu-anchored defective UiO-66 before and after H_2_ activation. Reproduced with permission from ref 23. Copyright 2019 American Chemical Society. (d) Synthesis of single Pt atoms anchored on porphyrin-based Al-MOF. Reproduced with permission from ref 27. Copyright 2017 Wiley-VCH GmbH.

In contrast to the destructive pyrolysis approach, the second strategy aims to retain the open-framework structure in MOF while incorporating SASs directly into it. However, these systems often involve additional complexities such as bimetallic sites, defect-rich sites, and unsaturated/open metal sites. Whether these resultant site configurations within MOFs can be rigorously classified as SASs requires careful case-by-case evaluation. To begin with, it is important to clarify the concept of unsaturated/open metal sites (OMSs) in MOFs. According to Kökçam-Demir et al., OMSs refer to coordinatively unsaturated metal sites that are created by removing terminal ligands through postsynthetic treatments without destroying the MOF.^21^ For instance, in our previous work, we constructed open Zn–N_2_ sites within ZIF-8 while preserving its SOD topology and porosity.^22^ Upon applying an electrochemical potential, these sites coordinate with hydroxide ions from the electrolyte to in situ form HO–Zn–N_2_ species (Figure 2b), which serve as the true active sites during the hydrogen evolution by promoting water adsorption and dissociation. It is clear that such OMSs differ fundamentally from the conventional definition of SASs; they are more akin to coordinatively unsaturated metal sites created by missing-ligand or missing-cluster defects rather than truly isolated and atomically dispersed metal sites. Moreover, to preserve the open-framework structures, it is impossible for all metal sites to exist as OMSs, and only a portion can be unsaturated. If we focus solely on the catalytically active OMSs, they could be tentatively regarded as SAS-like configurations due to their reduced metal density and increased metal–metal distance. However, when considering the coexistence of both saturated and unsaturated metal sites (such as Zn–N_4_ and Zn–N_2_ in ZIF system), it becomes difficult to classify all metal centers of the same element as SASs.

Building on this foundation, secondary metals can be further introduced to generate additional OMSs and/or defect sites within the preserved MOF structure, that serve as the true catalytically active sites, referred to here as MOF-type SACs. For instance, Abdel-Mageed et al. and Impeng et al. immobilized highly active Cu on defective UIO-66 (Zr) for catalytic reactions (Figure 2c).^23,24^ Similarly, Ren et al. employed a comparable strategy to deposit Ru single atoms onto UIO-66, establishing strong electronic metal–support interactions (EMSI) between the Ru SASs and the MOF framework.^25^ They utilized the defect nodes to stabilize the anchoring of secondary metals, achieving the low-loading metal substitution. In these cases, the intrinsic metals (Zr) are not active sites; rather, the catalysis originates from the deliberately secondary metals (Cu, Rh) acting as true active sites. Another approach involves the use of functionalized nitrogen-rich ligands, such as porphyrin, bipyridine-, and Salen-based ligands, which provide empty coordination sites for anchoring secondary metal SASs.^26^ A direct approach involves utilizing prefunctionalized macrocycles; for instance, Fang et al. anchored isolated Pt atoms within an Al-MOF through strong interactions with four N atoms of porphyrin ligand, obtaining Pt SASs with outstanding catalytic hydrogen evolution (Figure 2d).^27^ Alternatively, for ligands lacking intrinsic binding sites, postsynthetic grafting offers a flexible solution. As demonstrated by Ma et al., who employed surface −O/OH_*x*_ sites of Zr_6_-oxo clusters can be used to immobilize secondary metals (Ni, Co, Cu), thereby stabilizing SASs within the UiO-66.^28^ Streamlining this synthesis even further, Liu et al. used a facile one-pot method to bond single Pt atoms to the organic functional groups (−Br, −NH_2_, −I, and −H) of the benzene-1,4-dicarboxylate (BDC) ligand in UiO-66, which resulted in highly uniform and tailorable active sites and was also effective for MOF-5 (Zn), MIL-101 (Fe), NiBDC and CuBDC.^29^ Meanwhile, Sanati et al. utilized a mixed-linker strategy to generate robust Mn/Co-MOFs with abundant open metal sites, achieving industrial-scale stability for urea oxidation in seawater.^30^ In addition, directly replacing the intrinsic ligands in MOFs with functionalized ligands with metal-binding groups, such as Ir­(ppy)_2_(L) and Pt­(H_2_L)­Cl_2_ (L = 2,2′-bipyridine-5,5′-dicarboxylate), can to some extent lead to the formation of target metal SASs while maintaining the great structural integrity of the MOF structures.^31^ By adjusting the mixing ratio of these ligands, the distance between metal centers on the ligands can be conveniently tuned. For the aforementioned MOF-type SACs, if the contribution of the intrinsic metal is neglected, the main active sites (the secondary metals) can reasonably be regarded as SASs; they exhibit larger metal–metal distances and lower metal loadings compared to the inherent metals. These systems are not limited to mononuclear MOFs, as one can freely select suitable and stable MOF topologies, create defect/OMS structures and subsequently anchor highly active secondary metals as SASs.^11^

However, in reality, such bimetallic MOF-type SAC systems are often highly complex under electrochemical conditions. Assuming that only the secondary metal contributes to catalysis while the intrinsic metal remains entirely inactive is an idealized scenario. The coexistence of multiple metals can lead to changes in electronic structure, entropy stabilization effects, or other phenomena.^32^ In addition, the retention of the porous framework and organic ligands in MOFs decreases the SASs accessibility and limits electron transport efficiency. Compared to monometallic NC-type SACs, these systems are much more complicated, requiring careful consideration of MOF structural stability and the active sites evolution during long-term electrocatalysis.^33^

The catalytic applications of the above NC-type SACs are mainly focused on electrocatalysis, as their NC supports provide superior conductivity, favorable electronic structures such as sp^2^ carbon networks and π-conjugated systems, and structural stability due to the removal of insulating organic phases. In contrast, MOF-type SACs are predominantly used in photocatalysis, since retaining the MOF structure benefits light absorption, electron localization, and electron–hole recombination suppression, advantages that are almost absent in electrocatalysis. Nevertheless, we believe that MOF-type SACs still hold great potential in electrocatalysis. In particular, emerging two-dimensional conductive MOFs, featuring π–d conjugated coordination specifically (e.g., metal-bis­(dithiolene)^34^ or hexaiminotriphenylene^35,36^ families), offer a compelling strategy to circumvent the conductivity bottleneck. These architectures intrinsically facilitate efficient charge transport along the skeleton while preserving well-defined atomic sites, thereby bridging the gap between high porosity and conductivity without the need for destructive pyrolysis. Complementing these electronic advances, the interfacial engineering of pillared Co-MOF@NiMn-LDH nanocomposites, as recently demonstrated by Abazari et al., has emerged as a robust pathway to reinforce framework stability and prevent active site agglomeration.^37^ Such retention strategies encourage a revisiting of the role of MOFs, not merely as sacrificial templates but as functional and partially retained scaffolds. A key future challenge will be to integrate highly active SASs with the intrinsic functionalities of MOFs, thereby unlocking the potential synergistic effects between SASs and MOF architectures in electrocatalysis.

## Limitations of MOF-Derived SASs

The construction of well-defined SASs via MOFs has yielded remarkable electrocatalytic metrics, primarily driven by their maximized atomic utilization efficiency. This advantage is particularly pronounced when experimental currents are normalized to the number of active sites, yielding exceptional specific activity and turnover frequency (TOF) values. However, it is crucial to note that the characteristically low mass loading of SASs often artificially inflates these apparent metrics. As critically highlighted by Jeong et al., conventional SASs are constrained by intrinsic limitations, including compromised stability, restricted metal loading, predominantly oxidized electronic states, and the absence of ensemble sites.^38^ Beyond these inherent issues, MOF-derived SASs face other critical challenges associated with the MOF treatments and the precise SAS regulation, which have rarely been addressed in previous reviews. Two critical aspects deserve particular attention: (1) the destruction of organic ligands and/or MOF structures during the treatments and (2) the inherent difficulty in ensuring reproducibility of the active site spatial distribution in each synthesis.

The first issue primarily stems from the dominant synthetic strategy: destructive thermal treatment (e.g., calcination, sulfidation, or phosphidation). This “burn-and-sacrifice” synthesis is widely employed to generate isolated, highly active SASs typical of the NC-type catalysts discussed earlier. Despite the framework decomposition, the intrinsic structural diversity of MOF precursors still affords systematic control over the local coordination environment, allowing for the fine-tuning of coordinating atoms (e.g., N, S, or P), electronic structures, and the degree of graphitization.^39^ Nevertheless, this process inevitably destroys the meticulously designed framework architecture. Using MOFs merely as sacrificial templates for anchoring SASs neglects their intrinsic advantages, such as tunable porosity, high surface area, and ordered crystallinity. This approach essentially reduces a sophisticated coordination polymer to a disordered carbonaceous shell, sacrificing the precise structural tailorability that distinguishes MOFs from conventional carbon supports. In addition, this SASs synthesis typically requires prolonged high temperature under inert gas protection, consuming large amounts of energy while yielding less than 5% of the final product. Complete evaporation of intrinsic metals (such as Zn) and the introduction of highly active noble/transition metals as secondary components contradict the obvious advantage of SAS design, namely, minimizing the use of precious metals widely employed in electrocatalysis.^38^

Although MOF-type SACs within preserved open-framework structures can mitigate some of the above issues, as illustrated by several examples earlier, these systems introduce additional complications. In particular, the identification and quantification of the true active sites, which are crucial for understanding the electrocatalytic mechanism, often become ambiguous due to the presence of bimetallic sites and complex porous architecture. Moreover, most MOF nanoparticles with 3D topologies are difficult to integrate into practical electrochemical devices, such as Membrane Electrode Assemblies (MEAs). In laboratory setups using a three-electrode cell, binders like Nafion are typically used to drop-cast or deposit MOF-type SAC powders onto electrode surfaces, which not only blocks the accessibility of active sites and pore channels but also poses a risk of detachment during reactions. In-situ growth or chemical vapor deposition (CVD) methods can effectively overcome these issues; Note that resulting crystal structures may differ from conventionally synthesized powders. In contrast, MOF-type SACs have demonstrated remarkable performance and widespread application in photocatalysis, benefiting from the intrinsic MOF advantages and their ability to be directly used as powder catalysts with sacrificial agents, rather than on electrodes surface. Combined with the inherent instability of SASs, the low conductivity resulting from organic ligands, and the tendency of MOFs to undergo metal leaching, the practical use of such electrocatalysts remains limited, mostly confined to preliminary laboratory-scale studies. It is crucial to note that such degradation often stems from intrinsic chemical instability, where electrolyte components such as pH or coordinating ions trigger ligand hydrolysis.^40^ This represents a fundamental limitation distinct from electrochemical corrosion that we have detailed elsewhere.^33^ Beyond these scientific hurdles, the economic and practical viability remains a formidable challenge.

The second limitation concerns the reproducibility of SAS synthesis. As observed in MOF defect engineering, it is inherently difficult to ensure that defects form at identical locations and concentrations in each synthesis.^41^ This issue similarly affects SASs anchored through OMSs or defect sites, where achieving consistent site density and spatial distribution across different synthesis batches is challenging. Although templating methods can to some extent generate well-defined and regular defect structures, the actual distribution of anchored SASs is largely influenced by the amount of metal precursor introduced and the post-treatment strategy. For instance, Guo et al. employed a SiO_2_-templated strategy to obtain single-atom Zn sites within nitrogen-doped carbon evolved from ZIF-8.^42^ Although the resulting material exhibited hierarchical and ordered porous carbon polyhedrons, the Zn SASs appeared well distributed in the center but more densely aggregated toward the edges in the HAADF-STEM image. Thus, the metal atoms are not always uniformly or completely anchored at all the engineered defect structures. Currently, most studies ambiguously describe SASs as “uniformly dispersed,” even though it is difficult to ensure and verify that their spatial positions remain roughly consistent from one synthesis to another. When multiple metals are incorporated into the MOF and high-temperature pyrolysis is involved, the system complexity increases further. Metal aggregation or separation may occur under different thermal conditions, making it even more challenging to precisely control and reproduce the formation of target SASs, requiring in situ environmental electron microscopic study.

## Characterization Techniques and Their Limitations

The key to characterizing SASs lies in demonstrating both the atomic isolation of metal sites and their high utilization efficiency. In essence, characterization should confirm that the observed electrocatalytic activity originates from these isolated metal sites rather than from small clusters or larger nanoparticles. Advanced techniques such as high-angle annular dark-field scanning transmission electron microscopy (HAADF-STEM) and synchrotron-based X-ray absorption fine structure (XAFS) spectroscopy are widely used to provide evidence of metal–metal distance, yet both methods suffer from frequent misuse and inherent limitations.

HAADF-STEM allows the direct visualization of individual bright atomic spots in real space, because the Z value of SASs differs from that of the support atoms. For instance, Lim et al. obtained HAADF image showing uniformly distributed ultrasmall bright dots with sufficient distances, suggesting formation of atomically dispersed Pt single sites (Figure 3a).^43^ However, the obtained images typically represent only local regions, and relying solely on STEM does not accurately reflect the overall coordination environment or statistical uniformity of SASs.^44^ Thus, selecting visually uniform areas to claim homogeneous atomic dispersion is unreliable, and must be complemented by other characterization techniques. For MOF-type SACs that retain part of their 3D topological framework, the final HAADF image represents a 2D projection of a 3D structure, meaning that SASs may be obscured or overlapped by the framework in certain orientations, creating a false impression of the presence or absence of atoms. Furthermore, the qualitative and quantitative capability of elemental analysis is limited, especially in systems containing multiple metals, where the energy-dispersive X-ray spectroscopy (EDS) signal of the target metal atoms is often extremely weak and noisy. Also note that the high electron-beam dose required for imaging can induce structural evolution of SASs, including aggregation or further dispersion. This issue is particularly critical for beam-sensitive MOFs and MOF-derived SASs, where such transformations are often unavoidable.^45^ Low-dose or cryogenic TEM can mitigate beam-induced damage to some extent, though usually at the expense of image resolution.^46,47^

**Figure 3:**
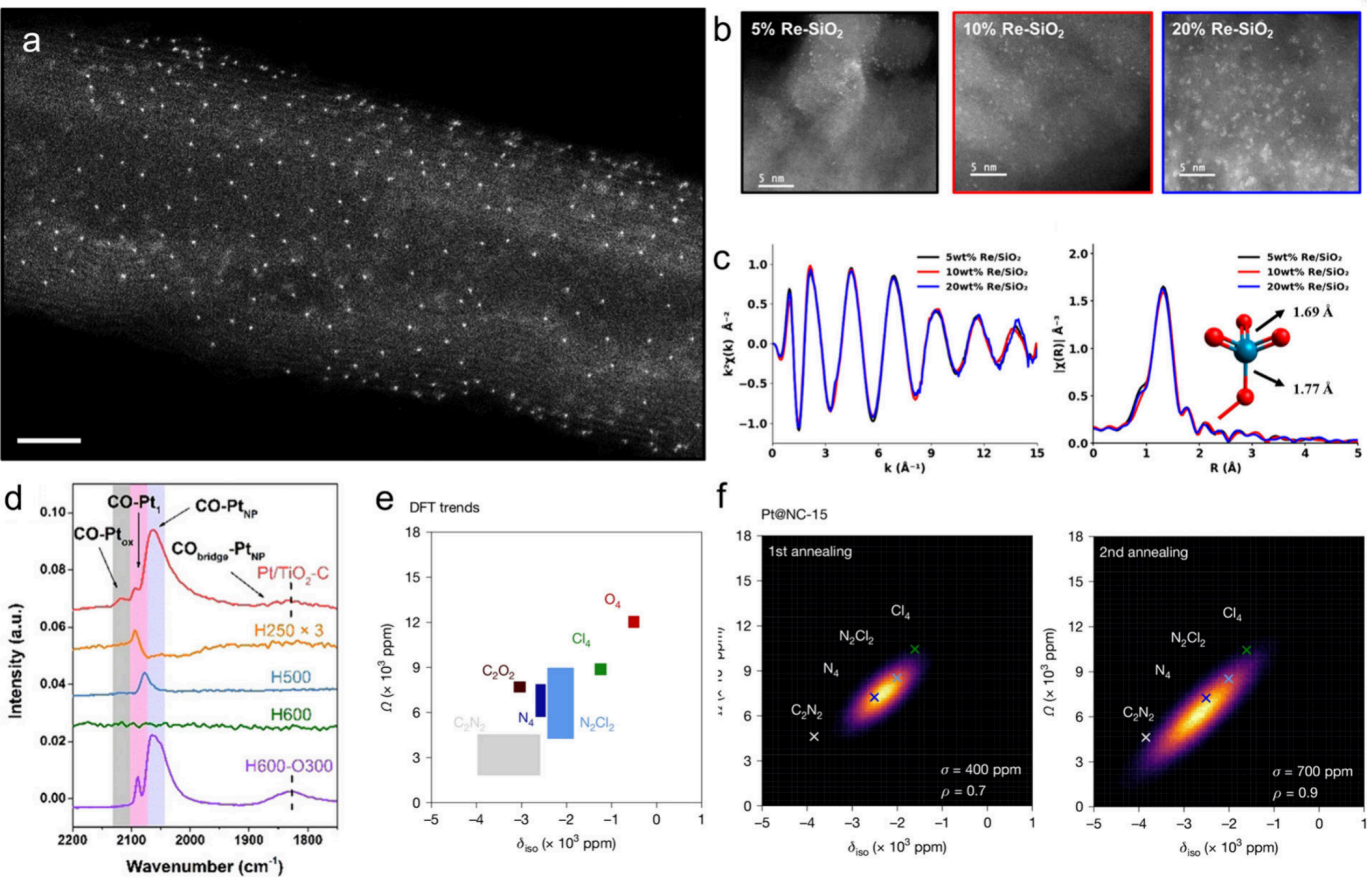
Advanced characterization of SASs. (a) HAADF-STEM image of Pt1/CNT catalyst showing isolated metal atoms. Scale bar: 3 nm. Reproduced with permission from ref 43. Copyright 2020 Springer Nature. (b) HAADF-STEM images and (c) *k* ^2^-weighted χ­(*k*) EXAFS spectra of Re/SiO_2_ with increasing metal loadings (5, 10, and 20 wt %). Reproduced with permission from refs 8 and 56 . Copyright 2020 and 2023 American Chemical Society. (d) In-situ CO–DRIFT spectra of Pt/TiO_2_ identifying specific adsorption sites. Reproduced with permission from ref 54. Copyright 2020 Wiley-VCH GmbH. (e) Computational modeling of NMR chemical shifts for different Pt coordination environments and (f) corresponding simulated δ_iso_–Ω-distribution maps for Pt/NC catalysts. Reproduced with permission from ref 55. Copyright 2025 Springer Nature.

In XAFS analysis, the extended X-ray absorption fine structure (EXAFS) region is Fourier transformed (FT) to obtain FT-EXAFS spectra as pseudodistance space, from which coordination environments (such as the identity, number, and distance of neighboring atoms) can be extracted through fitting.^48^ The evaluation of average metal–metal distances in a sample mainly relieson the analysis of FT-EXAFS spectra. In simple terms, most studies assess the presence of SASs by examining whether signals corresponding to M–N, M–O, or M–S coordination appear in the first-shell region (∼0.5–2.5 Å) and whether potential M–M interactions are absent in the second-shell region (∼2.5–3.0 Å). Standard EXAFS fitting protocols are routinely applied to confirm primary coordination spheres; for instance, Jiao et al. analyzed FT-EXAFS spectra of MOF-derived Fe–Ni–N–C catalysts and attributed signals ∼1.5 Å to Ni–N/Fe–N coordination, while the absence of Ni–Ni/Fe–Fe interactions was taken as evidence of complete atomic dispersion.^49^ Such fitted EXAFS results provide average coordination information over the illuminated sample volume, and are typically complemented by local structure from HAADF images, together offering compelling evidence for the presence of SASs.

However, interpretation of the second coordination shell is often complex because the derived metal structures depend on fitting experimental data to assumed models. As several recent reviews have noted, statements such as “the absence of second-shell signals indicates the absence of metal–metal or an oxide phase” are imprecise and potentially misleading.^8,9,50^ A cautionary example highlighting this ambiguity was reported by Qi et al. reported atomically dispersed Re species on mesoporous SiO_2_, where HAADF-STEM images showed highly dispersed Re species at a 5 wt % loading but clear clustering at 20 wt % (Figure 3b).^8^ In contrast, the corresponding FT-EXAFS spectra showed no significant differences with increasing Re loading and lacked distinctive Re–Re signals at higher *R* distance (Figure 3c).^8^ This underscores the limited quantitative ability of FT-EXAFS to distinguish systems containing both single metal atoms and clusters, particularly in cases with M–O–M scattering of bulk oxides (20% Re-SiO_2_). Similar limitations arise in studies involving other noble-metal systems (Pt, Pd, Au), where EXAFS often underrepresents subtle clustering effects.^50–52^ In this regard, Finzel et al. further cautioned against overinterpreting EXAFS results in MOF-calcined SAS systems.^8^ To resolve such ambiguities, comparing experimentally measured XAFS spectra with theoretical simulations offers a robust verification pathway. For instance, Lu et al. simulated the Ru K-edge XAFS spectra of Ru–NC–700, providing definitive evidence to distinguish between coexisting Ru single atoms and Ru nanoparticles.^53^ Furthermore, as emphasized by Chen et al., comprehensive analysis must go beyond spectral matching; EXAFS-derived parameters (specifically coordination number, bond length, and the Debye–Waller factor) hold critical diagnostic value in determining the precise SAS configuration and should be rigorously evaluated.^50^

For metals that exhibit strong CO binding affinity (e.g., Pt and Pd), CO–Diffuse Reflectance Infrared Fourier Transform Spectroscopy (CO–DRIFTS) serves as a powerful probe for vibrational features, offering insights into atomic structure and coordination geometry. For instance, Han et al. identified a band position at 2096 cm^–1^ in the CO–DRIFTS spectrum of Pt/TiO_2_, which, based on the fitting analysis, was attributed to linearly adsorbed CO on single Pt atoms (Figure 3d).^54^ In contrast, the clusters or nanoparticles typically generate absorption bands corresponding to bridge-bonded CO, which requires two adjacent metal atoms, appearing at about 1750–1950 cm^–1^. CO molecules can readily diffuse into MOF pores without causing structural disruption, selectively adsorbing onto the truly accessible metal sites, an aspect that is often difficult to achieve using XAFS or STEM alone. Thus, for MOF-derived SASs of specific metals, the position of the CO adsorption bands can serve as an effective indicator for distinguishing isolated SASs from clusters or nanoparticles. Beyond conventional methods, element-selective Nuclear Magnetic Resonance (NMR) spectroscopy has proven particularly effective for probing the local environment of metal centers. Koppe et al. employed static and ^195^Pt ultrawideline NMR methodologies, combined with simulations of square-planar Pt­(II) sites, to rigorously confirm the conversion of the H_2_Pt­(IV)­Cl_6_ precursor in ZIF-8 into Pt/NC SASs (Figure 3e).^55^ By analyzing the δ_iso_–Ω-distribution maps derived from calculated NMR lineshapes, they unveiled the heterogeneity of the chemical environment and local geometry of the Pt sites (Figure 3f). After the first annealing step, the Pt­(II) sites remain dominated by Cl/N-mixed ligand set, while a change to N-dominated ligand set after the second annealing. This methodology also enables the quantitative monitoring of coordination evolution during both synthesis and catalytic operation.

Complementary characterization techniques can more accurately verify SAS configurations, since each method has its own advantages and limitations. Besides the characterizations discussed above, other techniques commonly applied to SASs include X-ray photoelectron spectroscopy (XPS), X-ray diffraction (XRD), Mössbauer spectroscopy and ultraviolet visible (UV–vis) spectroscopy. The integration of specially designed electrochemical cells, which couples reactivity testing with a comprehensive suite of ex situ, in situ, and operando characterizations, constitutes the state-of-the-art protocol for elucidating the structural evolution of SASs. Operando/in situ techniques (particularly XAFS, infrared and Raman) enable the real-time monitoring of dynamic changes in the electronic structure and coordination environment of both SASs and their MOF supports, capturing critical transformations during synthesis (e.g., pyrolysis) and electrocatalytic reactions. As we have emphasized in our previous perspective, the MOF structural evolution under electrocatalytic conditions is nearly unavoidable and should be harnessed rather than ignored.^33^ By regarding the pristine MOF as a “pre-catalyst,” one can utilize the electrochemical restructuring process to in situ generate thermodynamically favorable active species (e.g., metal oxyhydroxides or defect-rich clusters). Therefore, the characterization methodologies are essential for precisely controlled SAS configurations within MOF structures and for understanding their structure–function relationships, which are of great importance for the future development of MOF-derived SASs.

## Accurate Evaluation of the SASs Utilization Efficiency

The second feature of SASs involves their theoretically high (often claimed 100%) atomic utilization efficiency; yet, systematic kinetic analysis validating this intrinsic advantage remains conspicuously absent from most electrocatalysis literature. The catalytic performance is typically evaluated by overpotential and current density. However, the widely used current density normalized by geometric area (mA cm^–2^) is strongly influenced by the electrode surface structure and therefore fails to represent the intrinsic SAC activity (Figure 4a).^57,58^ This issue is particularly pronounced when using porous carbon cloth or metal foam substrates with large surface areas as the working electrode of SACs. The normalization of current density by catalyst loading (A g^–1^) presupposes that all active sites within the loaded SAC are fully accessible to the electrolyte and actively participate in the reaction.^59,60^ To address this uncertainty, high-precision electrocatalysis studies increasingly prioritize specific activity and turnover frequency (TOF) as more robust indicators of intrinsic performance. While specific activity normalizes the current to the electrochemically active surface area (ECSA), the calculation of TOF necessitates the precise quantification of the actual number of active sites, as outlined in the following equations:^60^

$$\begin{array}{l} j_{\mathrm{Norm}}=\frac{I}{A_{ECSA}}\\ TOF=\frac{j\times N_{a}}{n\times F\times \Gamma_{mol}} \end{array}$$

where *j*, *n*, *N*_a_, *F*, and Γ_mol_ represent the current density, the number of electrons transferred, the Faraday constant, and the surface molar density of active sites, respectively. These calculations underscore that the precise quantification of active site density is a prerequisite for accurately evaluating the intrinsic activity of SACs. Crucially, this metric also serves as the benchmark for validating atomic utilization efficiency. While near-100% utilization is often cited as a theoretical hallmark of SASs, substantiating that every metal atom actively participates in the reaction requires rigorous kinetic verification. Common experimental methods used to estimate ECSA and/or active-site density include the double-layer capacitance (*C*_dl_) method, H/Cu/Pb-underpotential deposition (UPD), CO stripping, and redox-peak integration.^59^ Among these, the *C*_dl_ method is most widely used to estimate the ECSA of metal-based electrocatalysts by dividing the measured capacitance by the specific capacitance (*C*_s_) of the material, as in the following equation:

$$ECSA=\frac{C_{dl}}{C_{s}}$$

Then, the measured current is normalized by the obtained ECSA to yield the specific activity. For instance, Zhai et al. evaluated the ECSA of catalysts using *C*_dl_ method, and found that NiMoN/NiFe LDH exhibited a much higher *C*_dl_ value than other samples, indicating a greater number of exposed catalytic active sites (Figure 4b).^61^ Furthermore, the ECSA-normalized current density revealed that the enhanced electrocatalytic activity was not only attributed to the enlarged ECSA but also to its promoted intrinsic activity of the metal sites (Figure 4c). However, despite its simplicity and popularity, the *C*_dl_ method has significant limitations when applied to SASs. It measures the nonfaradaic current arising from ion adsorption and desorption on the electrode surface, reflecting the geometrically accessible area or overall charge storage capability of the conductive surface rather than the true number of catalytic active sites. Anantharaj et al. also highlighted that the *C*_s_ value, which is a key parameter in calculating ECSA, varies depending on surface properties and electrolyte environments.^60^ However, many studies that directly use reported or literature *C*_s_ values to calculate ECSA may yield inaccurate results. In MOF supports or MOF-derived SACs, extensive conductive but catalytically inert surface regions (e.g., intrinsic metals, sp^2^ carbon, oxygen-containing groups) contribute to the measured *C*_dl_ yet do not participate in catalysis. Since SASs constitute only a minute fraction of the total surface, a higher *C*_dl_ (and thus higher ECSA) does not necessarily indicate a greater number of active sites or higher metal utilization.

**Figure 4:**
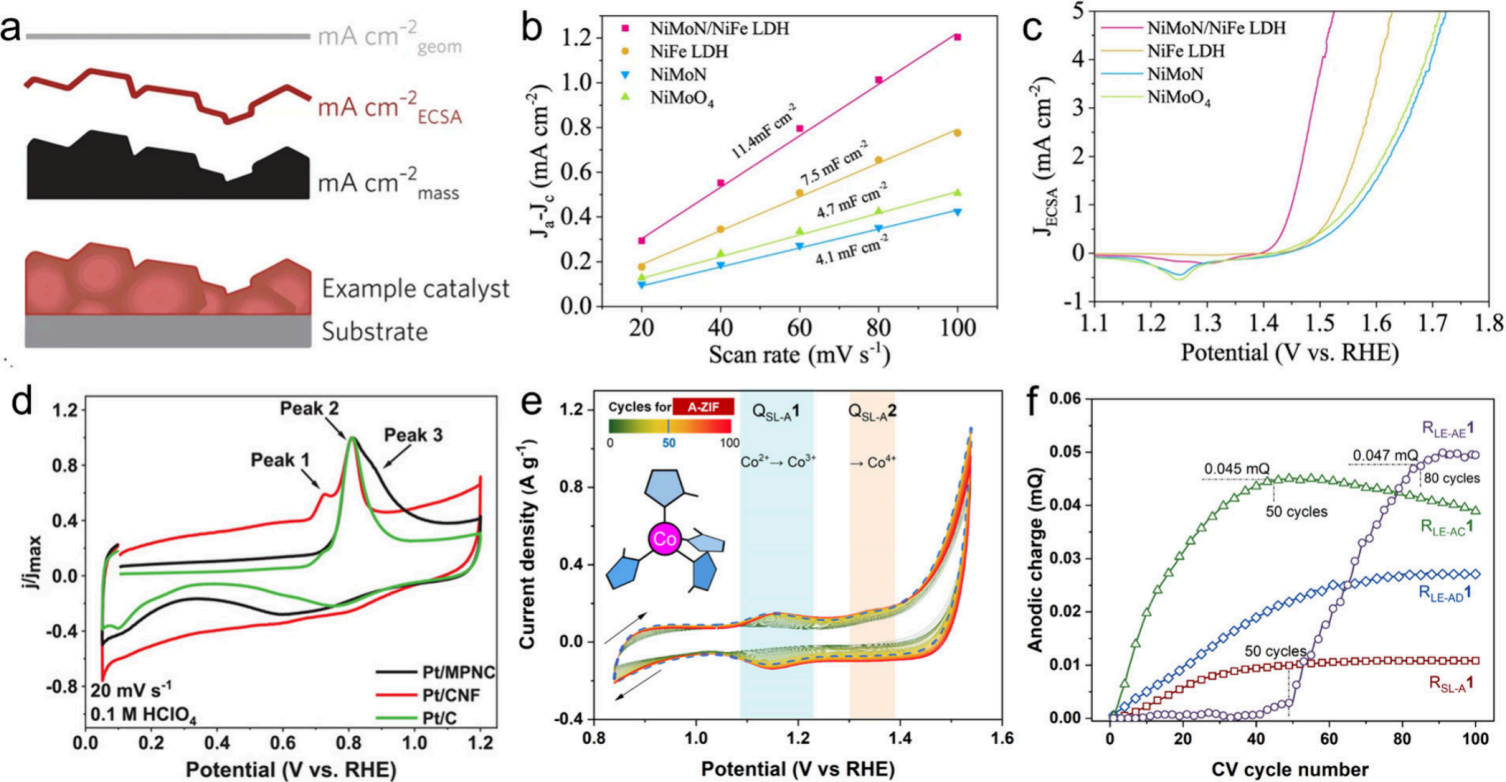
Evaluation of atomic utilization efficiency. (a) Schematic comparison of different activity normalization metrics: catalyst mass (black), electrochemically active surface area (ECSA, red), and geometric area (gray). Reproduced with permission from ref 58. Copyright 2017 Springer Nature. (**b**) Electrochemical *C*_dl_ determination and (c) ECSA-normalized polarization curves distinguishing intrinsic activity. Reproduced with permission from ref 61. Copyright 2022 Springer Nature. (**d**) CO-stripping voltammograms for quantifying active sites. Reproduced with permission from ref 63. Copyright 2023 Wiley-VCH GmbH. (e) Evolution of CV curves of A-ZIF (Co-ZIF-67) and (f) anodic charge integration of a series of ligand-engineered ZIF-67, illustrating the dynamic reconstruction and active site accessibility. Reproduced with permission from ref 66. Copyright 2024 Springer Nature.

In contrast, the latter three methods, UPD, CO stripping, and redox-peak integration, offer more accurate quantification of active metal sites and are thus better suited for calculating TOF. Nonetheless, their applicability remains limited, as they cannot be universally applied to all metal-based electrocatalysts. For CO stripping, the active surface area is quantified by integrating the anodic charge transferred during the oxidative removal of a saturated CO monolayer chemisorbed on the metal surface.^62^ This method offers high selectivity for sites with strong CO-binding affinity (e.g., Pt, Pd, Ni) and the CO adsorption–oxidation sequence mirrors key elementary steps in electrocatalysis. The number of electrochemically active sites (*n*_active_) can be quantified from the CO stripping charge (*Q*_CO_) using the following equation:

$$n{\left(mol\right)}_{active}=\frac{Q_{CO}}{nF}$$

where *Q*_co_, *n*, and *F* represent the integrated charge obtained from CO stripping, the number of electrons transferred during CO oxidation (typically 2 e^–^ per oxidized CO molecule, CO + H_2_O → CO_2_ + 2H^+^ + 2e^–^), and the Faraday constant, respectively. Zeng et al. employed CO stripping in 0.1 M HClO_4_ to reveal Pt single atoms, clusters, and nanoparticles and further calculated their corresponding ECSA_CO_ by integrating the CO oxidation charge (Figure 4d).^63^ For internal SASs located within MOF micropores that are smaller than CO molecules (∼3.8 Å), CO access is limited, resulting in weak or unobservable adsorption signals and thus an underestimation of the number of truly active sites. CO stripping requires the metal center to transfer electrons to the electrode substrate, which may be hindered in low-conductivity MOFs, leading to sluggish CO stripping kinetics or shifting onset potentials, which complicates the accurate integration of charge. Moreover, CO stripping is strongly influenced by the crystallographic facets of the metal, a limitation also shared by the UPD method, which typically uses hydrogen or other metal ions in the electrolyte as probes.^60,64,65^

Alternatively, redox-peak integration derives ECSA by quantifying the charge associated with specific reversible redox transitions in cyclic voltammetry (CV). This method exploits the direct proportionality between the integrated Coulombic charge and the population of electrochemically accessible surface atoms. It is worth noting that unstable SASs and its MOF structures may exhibit significant variations in oxidation peak shape during continuous CV cycles, thereby affecting the accuracy of integration. For instance, in our previous study on ZIF-67 (Co), both the position and area of the oxidation peak changed markedly after prolonged CV cycling (Figure 4e). This resulted in a substantial increase in the number of electron-accessible Co sites after 100 CV cycles compared with the first cycle (Figure 4f).^66^ In addition, when subtracting the background current, careful selection of the baseline is crucial, as separate baseline measurements can improve the accuracy of the methods involving integration.

The choice of quantification method should be tailored to the metal–support system, with cross-validation often required to determine the exact density of electrochemically active SASs. Once the active site population is accurately quantified, specific activity and TOF can be calculated, thereby enabling a true assessment of the intrinsic electrocatalytic efficiency independent of geometric loading. Then, the total amount of the target metal in the catalyst layer (at electrode loading) can be determined through elemental analysis such as Inductively Coupled Plasma (ICP) techniques, yielding the weight percent, *w*_m_. The total number of metal-based SASs in the catalyst layer, *n*(mol)_total_, can then be calculated using the following equation:^43^

$$n{\left(mol\right)}_{total}=\frac{w_{M}\times \rho_{cat}\times V_{cat}}{100\times M_{M}}$$

where *M*_M_ is the metal molar mass, ρ_cat_ is the mass loading of the catalyst powder on the electrode and *V*_cat_ is the volume of the loaded catalyst ink, and *w*_M_ is the weight percentage of the metal in the bulk catalyst. By comparing the total number of metal sites (*n*_total_) on the electrode with the number of electrochemically active sites (*n*_active_) in the reaction, the metal utilization efficiency of SACs can be determined by the following equation:

$$\mathrm{Utilization}\;\left(\%\right)=\frac{n{\left(mol\right)}_{\mathrm{active}}}{n{\left(mol\right)}_{\mathrm{total}}}\times 100$$

This calculation is crucial for evaluating the metal utilization efficiency of SASs, as it helps verify whether all the metal-based SASs on the electrode actually participate in the catalytic reaction. This is particularly important for SACs with complex MOF pore structures, for understanding the reaction mechanism and the SAS accessibility. If the calculated utilization approaches 100%, excluding experimental uncertainties, it can be assumed that all SASs anchored within the open-framework structure (including the interior) actively serve as catalytic sites. In contrast, if the utilization is below 40%, the catalytic process is likely limited to surface sites, with internal sites in inaccessible pores remaining inactive. At present, the utilization value of MOF-derived SACs is strongly influenced by factors such as the pore size and the electrode loading. Larger pore structures facilitate the exposure of SASs to the electrolyte and promote efficient gas release, thereby enhancing utilization. Often requires longer organic ligands, however, which affects the MOF structural stability during reactions. Therefore, optimizing MOF architectures and SAS synthetic strategies based on the calculated utilization results represents a promising direction for the future development of advanced MOF-derived SACs in electrocatalysis.

## Conclusion and Outlook

This Mini-Review underscores that the advancement of MOF-derived single-atom electrocatalysts hinges not merely on discovering new architectures, but on a paradigm shift toward rigorous methodological validation. The definition of SASs must evolve from a loose structural label to a quantitative metric of atomic utilization efficiency, validated by precise quantification of participating active sites rather than geometric surface area. While destructive pyrolysis remains dominant, we argue that the field must pivot toward framework-retaining strategies to reconcile the trade-off between electrical conductivity, porosity, and active site uniformity without sacrificing the intrinsic designability of the MOF architecture.

Looking ahead, the frontiers of atomic engineering extend well beyond mononuclear sites. The emergence of “dual-atom” or “triple-atom” moieties represents a strategic evolution, offering a unique opportunity to break the linear scaling relationships that often constrain single atoms.^67–69^ By engineering synergistic electronic coupling between adjacent metal centers, kinetic barriers for complex multistep reactions can be significantly lowered. However, similar to the limitations emphasized here for single-atom verification, ambiguous structural definitions and insufficiently conclusive characterization undermine the credibility of these more complex atomically dispersed configurations. Notably, the boundary between these species requires careful delineation, as metal clusters can sometimes outperform isolated atoms, as demonstrated by the superior activity of nanometer-sized Pt clusters in styrene hydrogenation.^70^ To realize the full potential of these complex configurations, preserving the open-framework structure of MOFs is critical. Unlike destructive post-treatments that obscure coordination environments, functionalized open frameworks serve as precise scaffolds for tracking structural evolution and maximizing the synergy between the MOF support and the anchored active centers.

Future efforts must integrate operando characterization with theoretical simulations to unveil explicit reaction pathways and adsorption energetics, thereby elucidating the dynamic structure–activity relationships of these complex active sites. Crucially, the static view of stability should be updated; we advocate harnessing dynamic reconstruction by treating MOFs as precatalysts that evolve into thermodynamically favorable active species in situ.^33^ To rigorously validate robustness, standard electrochemical testing must be complemented by postcatalysis forensics. Techniques such as ICP-MS and HAADF-STEM should be routinely employed to definitively exclude metal leaching and aggregation, ensuring performance stems from stable single sites. Furthermore, data-driven approaches are accelerating discovery; for instance, Kum et al. utilized machine learning to screen over 4000 Ru–SAC–MOF structures, showcasing the power of AI in navigating vast design spaces.^71^ Ultimately, bridging the gap to industrial relevance requires addressing economic sustainability, avoiding the waste of expensive ligands, and mastering the integration of these catalysts into Membrane Electrode Assemblies (MEAs) for scalable energy conversion.
